# Characterize the switching performance of a superconducting nanowire cryotron for reading superconducting nanowire single photon detectors

**DOI:** 10.1038/s41598-019-52874-3

**Published:** 2019-11-08

**Authors:** Kai Zheng, Qing-Yuan Zhao, Ling-Dong Kong, Shi Chen, Hai-Yang-Bo Lu, Xue-Cou Tu, La-Bao Zhang, Xiao-Qing Jia, Jian Chen, Lin Kang, Pei-Heng Wu

**Affiliations:** 1 Research Institute of Superconductor Electronics (RISE), School of Electronic Science and Engineering, Nanjing University, Nanjing, Jiangsu 210023 China; 2 School of Physics and Electronic Electrical Engineering, Huaiyin Normal University, Huai’an, Jiangsu 223003 China

**Keywords:** Superconducting devices, Superconducting devices, Superconducting devices, Superconducting devices, Characterization and analytical techniques

## Abstract

Scalable superconducting nanowire single photon detector (SNSPDs) arrays require cryogenic digital circuits for multiplexing the output detection pulses. Among existing superconducting digital devices, superconducting nanowire cryotron (nTron) is a three-terminal device with an ultra-compact size, which is promising for large scale monolithic integration. In this report, in order to evaluate the potential and possibility of using nTrons for reading and digitizing SNSPD signals, we characterized the grey zone, speed, timing jitter and power dissipation of a proper designed nTron. With a DC bias on the gate, the nTron can be triggered by a few μA high and nanoseconds wide input signal, showing the nTron was capable of reading an SNSPD pulse at the same signal level. The timing jitter depended on the input signal level. For a 20 μA high and 5 ns wide input pulse, the timing jitter was 33.3 ps, while a typical SNSPD’s jitter was around 50 ps. With removing the serial inductors and operating it in an AC bias mode. The nTron was demonstrated to be operated at a clock frequency of 615.4 MHz, which was faster than the maximum counting rate of a typical SNSPD. In additional, with a 50 Ω bias resistor and biased at 17.6 μA, the nTron had a total power dissipation of 19.7 nW. Although RSFQ circuits are faster than nTrons, for reading SNSPD or other detector arrays that demands less operation speed, our results suggest a digital circuit made from nTrons could be another promising alternative.

## Introduction

Superconducting nanowire cryotron (nTron) is a three-terminal device made from superconducting nanowires and it is capable of building superconducting digital circuits^1^. It has several advantages, such as ultra-compact size, large fan-out number, and insensitiveness to magnetic noise. In particular, an nTron has large output impedance and can drive a CMOS transistor directly^2^. It also has been demonstrated that an SFQ pulse can trigger an nTron, so does the output from a superconducting nanowire single-photon detector (SNSPD)^1,2^. Besides, nTrons have been used in some hybrid cryogenic circuits, such as driving a CMOS memory^3^, and driving LEDs in a photonic neuromorphic computer^4^.

The interfacing performance of the nTrons is well utilized, suggesting that nTrons are promising in reading signals from superconducting detectors. Although a RSFQ can have clock frequency up to 100 GHz, most superconducting detectors are much slower. Thus, the speed of SFQ circuits are not fully taken advantage of. For example, the SNSPD is single photon counting detector and its counting rate is typically less than 100 million counts per second^5^. Moreover, in an SFQ circuit, any input signal has to be digitalized to SFQ pulses before logic processing, and any output SFQ pulse has to be amplified to a level that room temperature electronics can read. For a superconducting computer, this signal incompatibility is not a severe problem since the amount of digital processing is heavy and most signals could be exchanged inside an SFQ chip. However, for other applications where non-SFQ signals are massively received and the amount of digital processing is moderate, for instance, a digital readout circuit for a superconducting detector array, signal-conversion circuits use much resources^6,7^.

Therefore, although nTrons are relatively slower than RSFQ circuits, we think they are promising in reading superconducting detectors, in particular for SNSPDs. Recently, developing large SNSPD arrays is a challenge, where a scalable and power efficient cryogenic signal readout is the key technology. Although some multiplexing readout schemes based on time domain or frequency domain have been demonstrated for reading moderate sized SNSPD arrays^8–10^, a focal plane detector array integrated with a compact and low power dissipation cryognic readout circuit would be a solution for achieving large scale SNSPD arrays. Since an nTron is also a superconducting nanowire device, it uses the same fabrication processes and can be integrated on a single chip with SNSPDs. Moreover, the switching area of an nTron is confined in a choke so that the integration with an nTron readout circuit can be very compact.

In this report, we proposed an nTron geometry and characterized its grey zone, speed, timing jitter and power dissipation. The experimental results are compared with the signal requirements of a typical SNSPD for evaluating the potential and possibility of using nTrons for reading and digitizing SNSPD signals. We also discussed tradeoffs between these parameters, from which the scalability, cooling budget, operation speed, etc. of a potential nTron digital circuit could be derived.

## nTron Geometry

**Figure 1:**
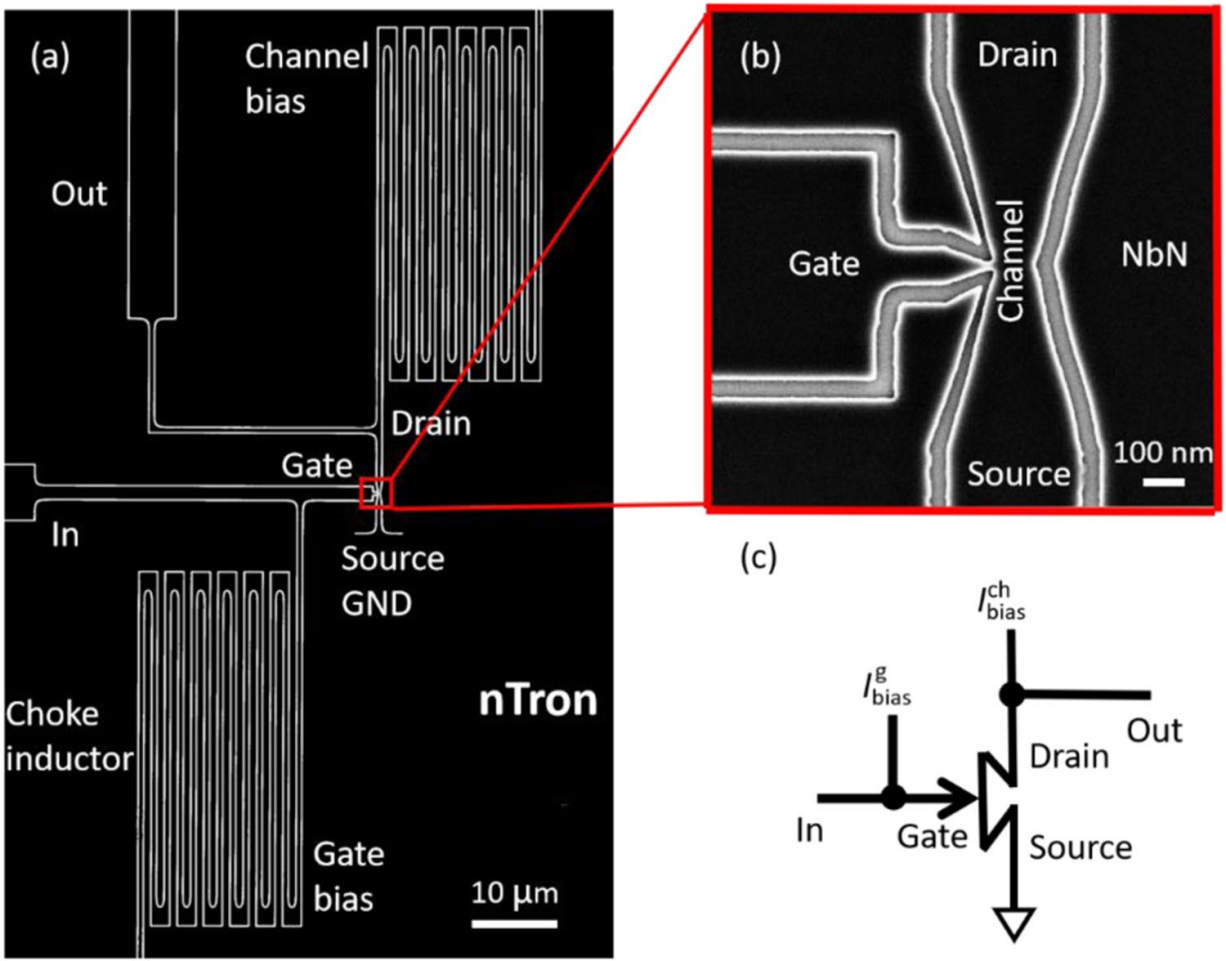
(**a**) Scanning electron microscope image of a typical nTron. An enlarged view of the choke area is shown in (**b**). (**c**) An equivalent circuit diagram for an nTron.

The nTrons were patterned from a ~10 nm-thick niobium nitride (NbN) film deposited on a thermal oxide silicon substrate. As shown in Fig. 1(a), it had a gate input port, a gate bias port, a channel output port, and a channel bias port. We fabricated devices of different geometries. To focus on the purpose of reading SNSPDs, in this paper the device analyzed was optimized in width and shape as shown in Fig. 1(a). Because a typical SNSPD made from SNSPDs has a width of 100 nm and a corresponding output current of 10~20 μA, we designed the width of the gate nanowire into 50 nm, which came out to be ~20 nm after fabrication. The width of the channel nanowire was chosen to be 100 nm considering the tradeoff between the sensitivity and gain. To maximize the current density around the choke area to let the nTron be triggered sensitively, as the SEM picture shown in Fig. 1(b), the channel was patterned into a bow tie shape. The widths and shape were fixed for the nTrons analyzed in this paper. The devices that had less fabrication constrictions and high critical currents were selected from current-voltage measurements and then chosen for switching measurements.

In this report, we characterized two nTron devices. Both of them used the same geometry. But one nTron had serial bias inductors and the other one had not. For the nTron used in grey zone measurements, the corresponding critical currents were 8.6 μA and 52.0 μA measured at liquid helium temperature. The meandered nanowires connected in series on the gate bias port and channel bias port acted as choke inductors to separate DC bias and RF output. Each bias port was connected in series with a 17.2 kΩ off-chip resistor. 

In the pulse measurements, we used an nTron without the serial inductors to minimize the overall inductance and replaced the bias resistors by 50 Ω resistors so that fast pulses can be applied with mimimum reflections. The fast nTron was fabricated on a thinner film. Therefore, the gate critical current reduced to 6.0 μA and the channel critical current was 21.9 μA. The input port and the output port each connected in series with an external 50 Ω resistor on a PCB to match the impedance of 50 Ω coaxial cables.

A circuit schematic diagram of the nTron is shown in Fig. 1(c), showing the bias, input and output ports. It is noticeable that, because the nTron has high output impedance, its output pulse can be read out directly after a room-temperature amplifier. Thus, we can prepare input pulses and get output signals off the nTron chip, enabling convenient characterizations without building another amplification stage.

## Threshold Currents, Grey Zone and Gain

**Figure 2:**
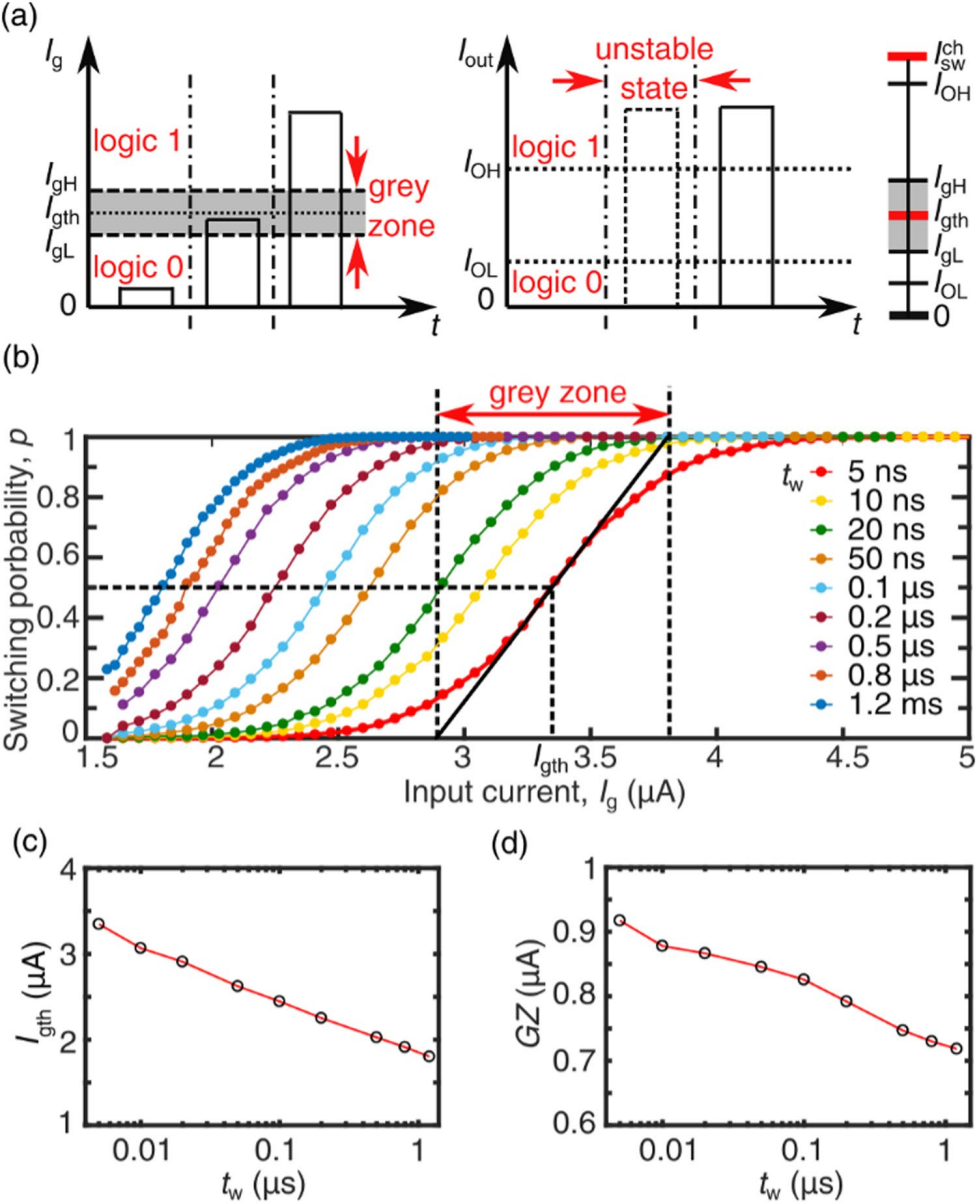
(**a**) A conceptual diagram to show the transient response of an nTron for different input currents, from which logic levels and grey zone can be visualized. The right bar shows logic values for an nTron. ${I}_{{\rm{s}}{\rm{w}}}^{{\rm{c}}{\rm{h}}}$ is the switching current of the channel, defining the maximum output current. *I*_OH_ and *I*_OL_ are the high logic level and low logic level for output, while *I*_gH_ and *I*_gL_ are the high logic level and low logic level for gate input. (**b**) Switching probability of the nTron versus the input curren*t I*_g_ at different pulse widths *t*_w_, while the channel bias and gate bias were fixed. (**c**) Threshold currents *I*_gth_ versus *t*_w_. (**d**) Grey zone values *GZ* versus *t*_w_.

An nTron operates like a digital comparator when it is used to read out SNSPD pulses. Since an nTron has current gain, it will amplify the SNSPD pulse into a large output pulse. We can take SNSPD pulse as digital input as well. When nTrons are used in building digital circuits, the switching behavior determines the logic levels and bias margin. As shown in Fig. 2(a), the minimum amplitude of the input current pulse for triggering an nTron channel *I*_gh_ defines the lower level for the input logic 1, while the maximum amplitude of the input current pulse for not triggering an nTron channel *I*_gL_ defines the upper level for the input logic 0. Between *I*_gL_ and *I*_gH_, there is an unstable region where an nTron switching stochastically due to thermal and quantum fluctuations^11^. This unstable region is referred to as grey zone^12^. Figure 2(b) shows the switching probabilities for the input pulse width *t*_w_ varying from 5 ns to 1.2 μs. For each point, we sent *N*_in_ = 5 × 10^5^ pulses to the gate and counted the number of switched pulses *N*_tr_. The switching probability was defined as *N*_tr_/*N*_in_. The channel bias was ${I}_{{\rm{bias}}}^{{\rm{ch}}}$ = 43.0 μA and the gate bias was ${I}_{{\rm{bias}}}^{{\rm{g}}}$ = 5.8 μA. We defined the pulse amplitude at switching probability *p* = 0.5 as the input threshold currents *I*_gth_. The grey zone of *GZ* was defined as *GZ* = 1/*ρ* where *ρ* was the slope of the switching probability curve at the input pulse amplitude equal to *I*_gth_.

With data shown in Fig. 2(b), we can extract the dependences of *I*_gth_ and *GZ* on input pulse width. As shown in Fig. 2(c), a shorter input pulse required a higher *I*_gth_. As an nTron channel’s output impedance was much higher than a typical 50 Ω load, the output current was proportional to its initial bias. With ignoring the leakage current through the channel, we can have the current gain of the nTron, which was $G=\frac{{I}_{{\rm{bias}}}^{{\rm{ch}}}}{{I}_{{\rm{gth}}}}$. Therefore, a shorter gate input pulse also resulted in a lower current gain and thus a lower fan-out number. At *t*_w_ = 5 ns, corresponding to a frequency of 200 MHz, *G* was 13.0. With a wider pulse, for instance, 1/*t*_w_ = 833 kHz, *G* increased to 23.9. We noticed that the *GZ* had a similar dependence on *t*_w_. Because there was noise adding on input pulses, when a wider pulse was applied, it was more likely to trigger an nTron, resulting in a narrower transition of the switching probability curve and a lower *GZ* value. Similar to what have been done on JJs, we can model the nTron switching dynamics with taking into account an energy barrier for escaping^11^.

## Timing Jitter

Low timing jitter is one of SNSPD’s advantages. Depending on the geometry, output signal-to-noise ratio, biasing current et. al, the timing jitter defined at full-width-at-half-magnitude varies from 3 ps to ~50 ps^13,14^. Thus, an nTron should have intrinsic low timing jitter so that the low timing jitter of an SNSPD read after an nTron digital circuit can be maintained. To study the switching jitter of the nTron shown in Fig. 1(a), we sent a 5 ns wide pulse into the nTron gate with varying the pulse amplitude and collected the delay between the input pulse and the nTron output pulse. The channel bias was ${I}_{{\rm{bias}}}^{{\rm{ch}}}$ = 43.5 μA and the gate bias was removed. Since the delay distributions were not all in a Gaussian shape, we used the root-mean-square value of the delay to define the RMS jitter. For each data point, more than 10^4^ pulses were collected by a high-speed oscilloscope for calculating the statistic values.

**Figure 3:**
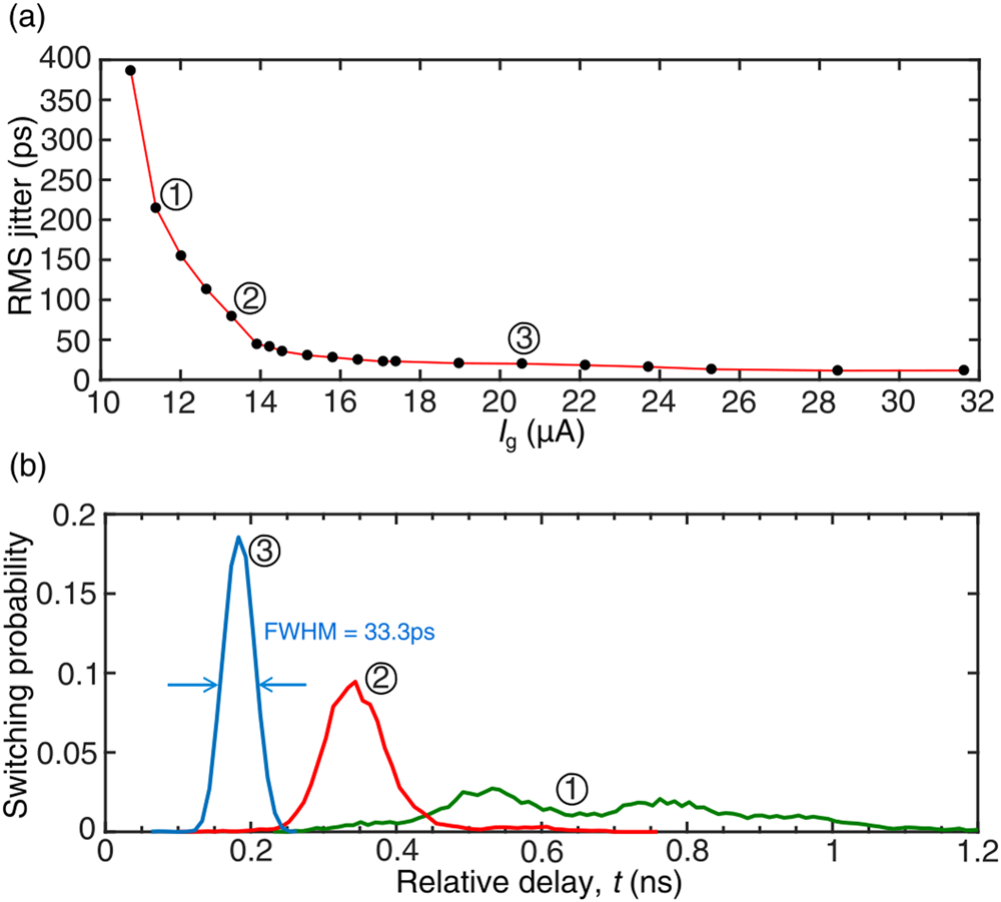
(**a**) The RMS Jitter vs. the input pulse amplitude *I*_g_. The pulse width was fixed at 5 ns. (**b**) Switching probability normalized to the total counts at three different input levels marked at (**a**) for *I*_g_ = 11.4 μA, 13.3 μA and 20.6 μA.

Figure 3(a) indicates the switching jitters for the input pulse amplitude varying from 10.7 μA to 31.6 μA. There was a clear decay of the jitter for a higher input pulse. As the current of input pulse increased, the jitter of an nTron trended to be stable. For instance, the RMS jitter at input pulse of 20.6 μA was 33.3 ps. We also observed the trigger delay became shorter for a higher input pulse. As shown in Fig. 3(b), when the input pulse increased from 13.3 μA to 20.6 μA, there was a 160 ps shift of the delay distribution. The profile of the delay distribution for high input pulses showed a Gaussian shape, while for low input pulse the distribution became wider and had multiple peaks, indicating a complex switching dynamics.

Both the timing jitter and the grey zone measurements indicated that the switching of an nTron started from probabilistic to deterministic as the input pulse increased. To understand such behavior of nTron needed a switching dynamic model with taking account fluctuations. In the first paper of nTron published in ref.^1^, Adam McCaughan and Karl Berggren suggested a 2D electro-thermal model, which could be used to simulate the switching dynamics for nTrons of different geometries. Since the nTron gate was 20 nm while the coherent length of NbN was about 10 nm, phase slippage and vortex dynamics in a nanowire can happen before a stable resistive domain was generated, which could be the sources for introducing wider grey zone and larger time jitter^15^. Based on the experimental results we have now, we cannot conclude a precise switching model for nTrons. However, the measured current threshold and timing jitter of the proposed nTron geometry can meet the requirement for reading an SNSPD-like pulse, i.e. 5 ns wide and 20 μA high.

## Operation Speed and Power Dissipation

The grey zone measurements showed that the nTron can be triggered by nanoseconds wide pulses, implying a possible operation speed of hundreds of MHz. To have a better characterization of the operation speed, i.e. the maximum clock frequency, of an nTron digital circuit, we measured the recovery time of a fast nTron. As nTrons were made from NbN nanowires, which had high kinetic inductance, current charging and discharging caused an electrical recovery time^16^. To reduce the electric recovery time, we removed the serial inductors on the bias ports, and shortened the nanowire to have less inductance. When nTrons were integrated on a single chip, connection length can be further reduced to speed up the electric recovery.

Another recovery time was from thermal relaxation of the local hotspot generated on a fired nTron. The hotspot was a resistive area where the local Joule heating increased the hotspot temperature. The power of input pulse can control the hotspot size and temperature. Thus, there was a tradeoff between the input signal power and the recovery time (i.e. the speed of nTron).

**Figure 4:**
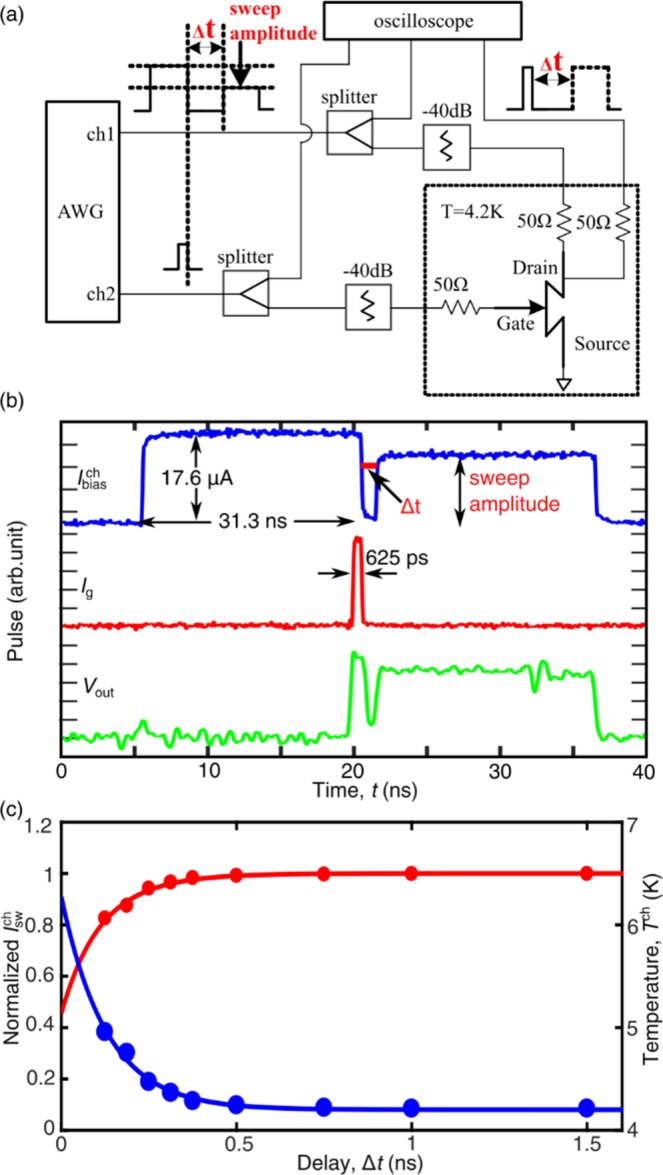
(**a**) Circuit diagram of the speed measurement setup. (**b**) Waveforms (blue for channel bias pulse; red for gate input pulse; green for output voltage pulse) for measuring the recovery time of an nTron. The nTron was fired at the first bias pulse. After a delay Δ*t* a second bias pulse was used to measure the time-dependent switching current of the channel ${I}_{{\rm{sw}}}^{{\rm{ch}}}(\Delta t)$. (**c**) Recovery of ${I}_{{\rm{sw}}}^{{\rm{ch}}}$ normalized to the static value ${I}_{{\rm{sw}}}^{{\rm{ch}}}$(4.2 K) (red trace) and recovery of the derived channel temperature *T* ^ch^ (blue trace).

To measure the thermal recovery time of an nTron, we implemented a pump-probe like measurement. As shown in Fig. 4(a,b), two bias pulses with a varied delay Δ*t* were sent to the nTron channel. The first bias pulse was 31.0 ns wide and 17.6 μA high. A gate pulse, which was 625 ps wide, was sent to trigger the nTron at the first bias pulse. We aligned the bias pulse and the input pulse to make them drop to zero simultaneously. Then, we sent a second channel bias pulse and swept its amplitude to find the switching current of the nTron channel of ${I}_{{\rm{sw}}}^{{\rm{ch}}}(\Delta t)$ as a function of Δ*t*. As we connected a 50 Ω resistor to the nTron gate to match the impedance of the coaxial cable, the input power sending to the nTron was ${P}_{{\rm{in}}}={I}_{{\rm{g}}}^{2}R$, where *R* = 50 Ω. Before the gate nanowire switched, the input power was dissipated mostly on the resistor. Once the gate nanowire switched into a normal resistor, the gate impedance increased much higher than 50 Ω. Thus, most power was dissipated on the nTron gate. Although some power was reflected, making the measurement of actual power dissipation of the gate nanowire difficult, by adjusting *P*_in_ we can control the maximum temperature that the gate nanowire reached after a switch, as shown in Fig. 4(c). The input power to the channel wire was calculated by the same method.

Figure 4(c) shows the recovery time of the nTron’s channel switching current after a switching. We converted ${I}_{{\rm{sw}}}^{{\rm{ch}}}(\Delta t)$ to *T*^ch^(Δ*t*) by solving Eq. (1)^17^, where *T*^ch^ was defined as an effective temperature of the channel with assuming a uniform temperature profile of the normal region of the channel.

1 $${I}_{{\rm{sw}}}^{{\rm{ch}}}={I}_{{\rm{c}}0}^{{\rm{ch}}}{[1-{(\frac{{T}^{{\rm{ch}}}}{{T}_{c}})}^{2}]}^{3/2}{[1+{(\frac{{T}^{{\rm{ch}}}}{{T}_{c}})}^{2}]}^{1/2}.$$

In Eq. (1), *T*_c_ = 8.1 K was the critical temperature of the NbN film and $\,{I}_{{\rm{c}}0}^{{\rm{ch}}}$ = 31.1 μA was the calculated critical current of the nTron channel at zero temperature. We fitted the data with an exponential decay ${T}^{{\rm{ch}}}={T}_{{\rm{\max }}}{e}^{-t/{\tau }_{{\rm{R}}}}$, where *T*_max_ was the maximum temperature at which *T*^ch^ started to drop and *τ*_R_ was the thermal relaxation time constant. When the input pulse current was 31.3 μA and the corresponding input power *P*_g_ was 48.9 nW, the calculated *T*_max_ was 6.3 K and the fitting thermal relaxation time *τ*_R_ was 0.13 ns.

**Figure 5:**
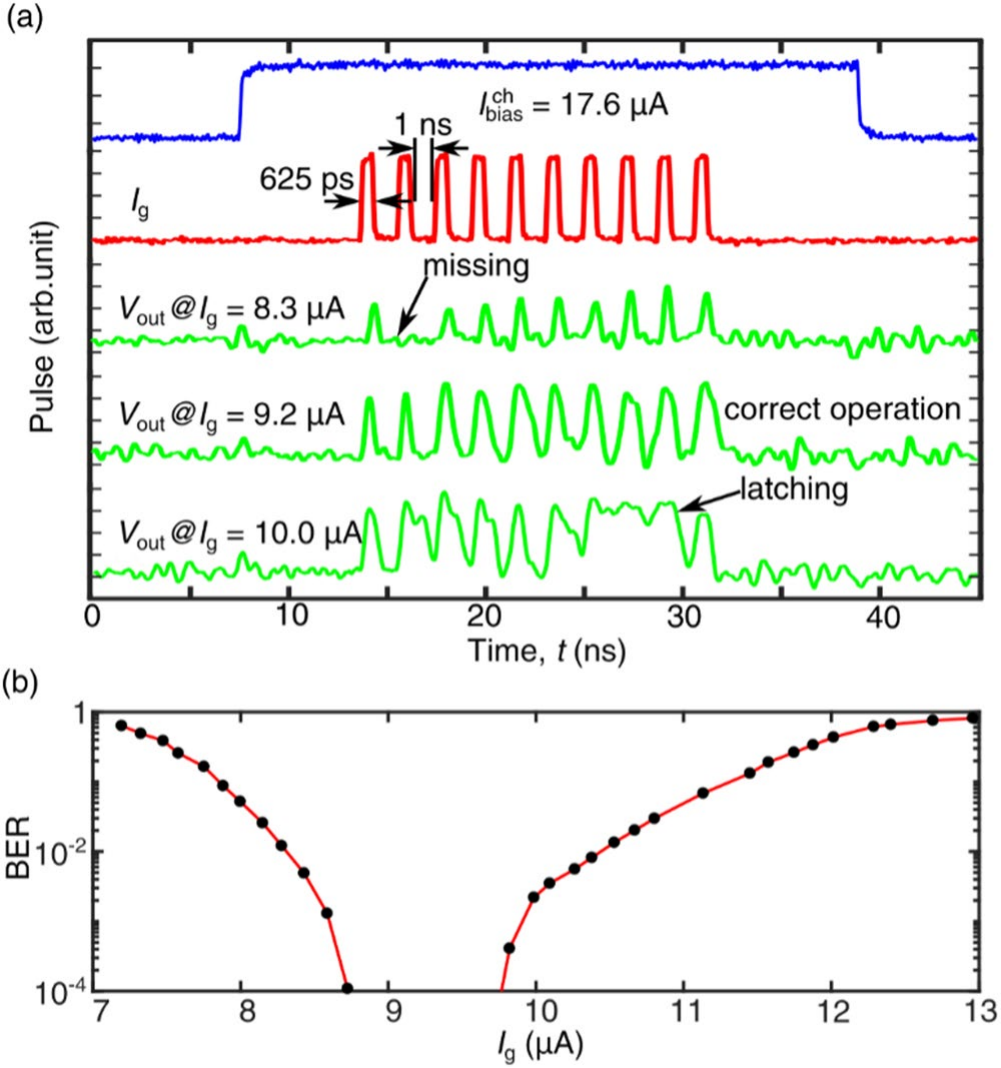
(**a**) Waveforms for operating the nTron at ten successive input pulses (red trace). The channel was biased with a long pulse (blue trace). The green traces are the output voltage pulses at three different inputs (*I*_g_ = 8.3 μA, 9.2 μA and 10.0 μA). (**b**) The bit error rate (BER) versus the input pulse amplitude *I*_g_.

From the above analyses, there was a tradeoff between the operation speed and the input power dissipation. If the input signal was high, although an nTron can be switched deterministically with low timing jitter, the nTron was heated up and needed a longer time for cooling, limiting the operation speed. If input pulses came with an interval time shorter than the relaxation time or the channel current recharged faster than the relaxation time, an nTron would latch. As shown in Fig. 5(a), we biased the nTron channel at 17.6 μA (0.8 ${I}_{{\rm{sw}}}^{{\rm{ch}}}$) and sent 10 successive input pulses at a frequency of 615.4 MHz. When the pulse amplitude *I*_g_ was too low, the nTron was not able to fire determinately and a few output pulses were missing. Nevertheless, if *I*_g_ was too high, we noticed that the output pulse sometimes latched because previous thermal relaxations had not fully completed. When *I*_g_ was set at a proper level, individual output pulses corresponding to each input pulse can be obtained. In Fig. 5(b), for each point, we collected 100k pulses and calculated the probability of errors. The Proper level of *I*_g_ can be gained when the bit error rate (BER) calculated by  the number of nTron output pulses divided by the total input pulse number was less than 10^−4^. We swept *I*_g_ and measured the bit error rate (BER) of the nTron output. As shown in Fig. 5(b), the margin of *I*_g_ for correct operations reduced to ~1 μA although the operation speed increased to 615.4 MHz and the gate input power was *P*_g_ = 4.2 nW at *I*_g_ = 9.2 μA. The channel was biased at 17.6 uA through a 50 Ω resistor. Thus, the input power to the channel wire was 15.5 nW. The total power sending to the nTron was 19.7 nW.

## Conclusions

In conclusion, we investigated the threshold currents, grey zone, speed and power dissipation of a typical nTron made from NbN films and measured at 4.2 K. We found those parameters related closely to each other and tradeoffs between them occurred. Experimentally, we demonstrated an nTron can be operated at a speed of 615.4 MHz with an input gate current of 9.2 μA and a total input power of 19.7 nW. The switching timing jitter of an nTron, included the measurement jitter caused by voltage noise, was 33.3 ps at a 5 ns wide and 20.6 μA high input pulse. Although the nTron’s performance are less superior to JJs in an advanced RSFQ circuit, the nTron we characterized in this work is faster enough to read a relatively slow superconducting detector, for instance an SNSPD that usually have a maximum counting rate less than 100 Mcps^18^. The nTron’s power dissipation is better than a cryogenic CMOS transistor, which usually dissipated mW power^19^. Thus, a mW cooling power could afford ~50 thousand nTrons, carrying out many digital circuits, e.g. a binary-coded decimal encoder or a counter.

It is worth to notice that the performance of an nTron depends on their design and geometry. If the size of the gate is much smaller, the gate critical current reduces so that the gate can be triggered at a low input current. Then, as the gate is narrow and thin, the local temperature of gate is higher with the same power dissipation on the gate. Meanwhile, an nTron made on substrates of high thermal conductivity, i.e. sapphire and MgO, will exhibit faster operation speed due to the short thermal recovery time. An nTron of wider channel can output more current, but the threshold current will increase as well. Understanding those dependences needs to study non-equilibrium dynamics of a superconducting nanowire, which is out of the scope of this work. Once the design of an nTron is made, characterization methods presented in work can be used for extracting its switching performance.

Recent works demonstrate that an nTron can be operated in a flux regime similar to shunted Josephson Junctions if we prevent the nanowire from heating to a hotpot^20–22^. This could give an nTron a considerable improvement in speed and power dissipation. However, as we mentioned in the introduction, an nTron operated in hotspot regime has its own advantages and is promising in certain digital applications. We wish the measured characteristics and tradeoffs shown in this letter can help researchers to design and utilize nTrons properly.

## References

[1] McCaughan AN, Berggren KK (2014). A superconducting-nanowire three-terminal electrothermal device. Nano Lett..

[2] Zhao Q, McCaughan AN, Dane AE, Berggren KK, Ortlepp T (2017). A nanocryotron comparator can connect single-flux-quantum circuits to conventional electronics. Supercond. Sci. Technol..

[3] Tanaka M (2017). Josephson-CMOS Hybrid Memory with Nanocryotrons. IEEE Trans. Appl. Supercond..

[4] Shainline JM, Buckley SM, Mirin RP, Nam SW (2017). Superconducting Optoelectronic Circuits for Neuromorphic Computing. Phys. Rev. Appl..

[5] Rosenberg D, Kerman AJ, Molnar RJ, Dauler EA (2013). High-speed and high-efficiency superconducting nanowire single photon detector array. Opt. Express..

[6] Miyajima S, Yabuno M, Miki S, Yamashita T, Terai H (2018). High-time-resolved 64-channel single-flux quantum-based address encoder integrated with a multi-pixel superconducting nanowire single-photon detector. Opt. Express..

[7] Ortlepp T (2011). Demonstration of digital readout circuit for superconducting nanowire single photon detector. Opt. Express..

[8] Zhao Qing-Yuan, Zhu Di, Calandri Niccolò, Dane Andrew E., McCaughan Adam N., Bellei Francesco, Wang Hao-Zhu, Santavicca Daniel F., Berggren Karl K. (2017). Single-photon imager based on a superconducting nanowire delay line. Nature Photonics.

[9] Doerner S (2017). Frequency-multiplexed bias and readout of a 16-pixel superconducting nanowire single-photon detector array. Appl.phys.lett..

[10] Hofherr M (2013). Time-tagged multiplexing of serially biased superconducting nanowire single-photon detectors. IEEE Trans. Appl. Supercond..

[11] Murphy A (2015). Three Temperature Regimes in Superconducting Photon Detectors: Quantum, Thermal and Multiple Phase-Slips as Generators of Dark Counts. Sci. Rep..

[12] Haddad T, Wetzstein O, Engert S, Toepfer H, Ortlepp T (2011). Investigation of the relationship between the gray zone and the clock frequency of a Josephson comparator. Supercond. Sci. Technol..

[13] Korzh, B. A. *et al*. Demonstrating sub-3 ps temporal resolution in a superconducting nanowire single-photon detector. Preprint at, https://arxiv.org/abs/1804.06839 (2018).

[14] Caloz M (2018). High-detection efficiency and low-timing jitter with amorphous superconducting nanowire single-photon detectors. Appl. Phys. Lett..

[15] Bae, M. H., Dinsmore, R. C., Sahu, M., & Bezryadin, A. Stochastic and deterministic phase slippage in quasi-one-dimensional superconducting nanowires exposed to microwaves. *New J. Phys*. **14** (2012).

[16] Kerman AJ (2006). Kinetic-inductance-limited reset time of superconducting nanowire photon counters. Appl. Phys. Lett..

[17] Il’in K, Siegel M, Semenov A, Engel A, Hubers H-W (2005). Critical current of Nb and NbN thin-film structures: The cross-section dependence. Phys. Status Solidi..

[18] Zhao Q (2014). Counting rate enhancements in superconducting nanowire single-photon detectors with improved readout circuits. Opt. Lett..

[19] Kerman AJ, Rosenberg D, Molnar RJ, Dauler EA (2013). Readout of superconducting nanowire single-photon detectors at high count rates. J. Appl. Phys..

[20] Shelly CD, See P, Ireland J, Romans EJ, Williams JM (2017). Weak link nanobridges as single flux quantum elements. Supercond. Sci. Technol..

[21] Brenner MW, Roy D, Shah N, Bezryadin A (2012). Dynamics of superconducting nanowires shunted with an external resistor. Phys. Rev. B.

[22] Toomey E, Zhao Q-Y, McCaughan AN, Berggren KK (2018). Frequency Pulling and Mixing of Relaxation Oscillations in Superconducting Nanowires. Phys. Rev. Appl..

